# Genetic Variants in ER Cofactor Genes and Endometrial Cancer Risk

**DOI:** 10.1371/journal.pone.0042445

**Published:** 2012-08-02

**Authors:** Yuqing Li, Hui-Qi Low, Jia Nee Foo, Hatef Darabi, Kristjana Einarsdόttir, Keith Humphreys, Amanda Spurdle, ANECS Group, Douglas F. Easton, Deborah J. Thompson, Alison M. Dunning, Paul D. P. Pharoah, Kamila Czene, Kee Seng Chia, Per Hall, Jianjun Liu

**Affiliations:** 1 Human Genetics, Genome Institute of Singapore, Singapore, Singapore; 2 Department of Medical Epidemiology and Biostatistics, Karolinska Institutet, Stockholm, Sweden; 3 Telethon Institute for Child Health Research, University of Western Australia, Western Australia, Australia; 4 Division of Genetics and Population Health, Queensland Institute of Medical Research, Brisbane, Queensland, Australia; 5 The Australian National Endometrial Cancer Study (ANECS) Group, Queensland Institute of Medical Research, Brisbane, Queensland, Australia; 6 Department of Public Health and Primary Care, University of Cambridge, Cambridge, United Kingdom; 7 Department of Oncology, University of Cambridge, Cambridge, United Kingdom; 8 Saw Swee Hock School of Public Health, National University of Singapore, Singapore, Singapore; Indiana University, United States of America

## Abstract

Given that the transcriptional regulatory activity of estrogen receptor (ER) is modulated by its biochemical cofactors, genetic variation within the ER cofactor genes may alter cellular response to estrogen exposure and consequently modify the risk for endometrial cancer. We genotyped 685 tagging SNPs within 60 ER cofactor genes in 564 endometrial cancer cases and 1,510 controls from Sweden, and tested their associations with the risk of endometrial cancer. We investigated the associations of individual SNPs by using a trend test as well as multiple SNPs within a gene or gene complex by using multi-variant association analysis. No significant association was observed for any individual SNPs or genes, but a marginal association of the cumulative genetic variation of the *NCOA2* complex as a whole (*NCOA2*, *CARM1*, *CREBBP*, *PRMT1* and *EP300*) with endometrial cancer risk was observed (P_adjusted_ = 0.033). However, the association failed to be replicated in an independent European dataset of 1265 cases and 5190 controls (P = 0.71). The results indicate that common genetic variants within ER cofactor genes are unlikely to play a significant role in endometrial cancer risk in European population.

## Introduction

Endometrial cancer is the most common gynecological cancer in Western countries and its incidence is increasing [1]. Biological, clinical and epidemiological studies have demonstrated that unopposed estrogen stimulates cell proliferation and induces carcinoma development of endometrial cancer [2], [3]. Estrogen plays its role in endometrial cancer development via the estrogen receptor by promoting its dimerization and translocation to the nucleus, where it modulates the expression of estrogen responsive genes [4].

Efficient transcription regulation by the estrogen receptor requires the participation of a class of proteins known as nuclear receptor coregulators, which consist of coactivators and corepressors [5], [6]. These coregulators play an important role in a variety of human diseases, including cancer and some metabolic disorders [7]. Nuclear receptor coactivators are rate-limiting in steroid receptor-mediated gene transcription [8]–[10] and have the ability to reverse the squelching of the transcriptional activity of one steroid receptor by another [9]. In addition to functioning as a bridge between receptors and the general transcriptional machinery, nuclear receptor coactivators act as cofactor complexes influencing receptor transcription regulation through a variety of mechanisms, including phosphorylation, acetylation, methylation, RNA splicing and chromatin remodeling [10], [11].

One family of coactivators, the P160 family is well-known to bind directly to hormone-activated estrogen receptors and recruit secondary coactivators [12], [13], where the *CREBBP* and the *CARM1* form an interaction domain that physically overlaps with the related transferable activation domains of the p160 family members [14]. Moreover, it is reported that a ternary complex formed by *NCOA2*, *CARM1* and *EP300* or *CREBBP* could enhance estrogen receptor (ER) function in an augmentation manner [15].

While the common genetic variation within hormone-related genes [16], [17] and the estrogen metabolism pathway [18] have been shown to be associated with endometrial cancer risk, the genetic variation of the ER cofactor genes, to our knowledge, has not carefully been examined for their involvement in the development of endometrial cancer.

In the current paper, we aimed to carry out a comprehensive association study of the polymorphisms within the ER cofactor genes with endometrial cancer risk. We conducted the discovery study in a population-based endometrial cancer sample of postmenopausal Swedish women and performed a validation analysis in an independent European dataset.

## Results

### Discovery Analysis

A total of 564 cases and 1510 controls were included in the discovery analysis (Table 1). All of the cases and controls were postmenopausal Swedish women. We successfully genotyped 685 tag SNPs within the 60 ER cofactor genes, which can capture 2410 common SNPs (MAF>5%) with 91% coverage in average (r^2^>0.8) according to the HapMap database. Out of these, 51 genes had coverage over 80%. Details of the SNP coverage evaluation is summarized in Table S1. In addition, we re-evaluated the coverage of same set of tag SNPs on variants MAF>5% in the 1000 genomes project database, which is an updated and more comprehensive catalog of human genome variation. The average coverage decreased to 64% and 19 genes had coverage over 80%. However, with r^2^>0.8, 5084 SNPs had been captured among 7141 SNPs in the 60 genes.

**Table 1:** Endometrial cancer sample sets and sources included in initial and validation analysis.

Study	Source	Cases	Controls	Genotyping platform
Discovery analysis				
Swedish Endometrial Cancer Study	Population based case-control study in Swedish postmenopausal women	564	1510	Illumina GoldenGate
Validation analysis				
Australian National Endometrial Cancer Study	Population based case-control study in Australia	599		Illumina 610 K
Study of Epidemiology and Risk Factors in Cancer Heredity	Population based case-control study in England	666		Illumina 610 K
Wellcome Trust Case-Control Consortium	Sample from 1958 Birth Cohort and UK Blood Donors from NBS		5190	Illumina 1.2 M

**Figure 1:**
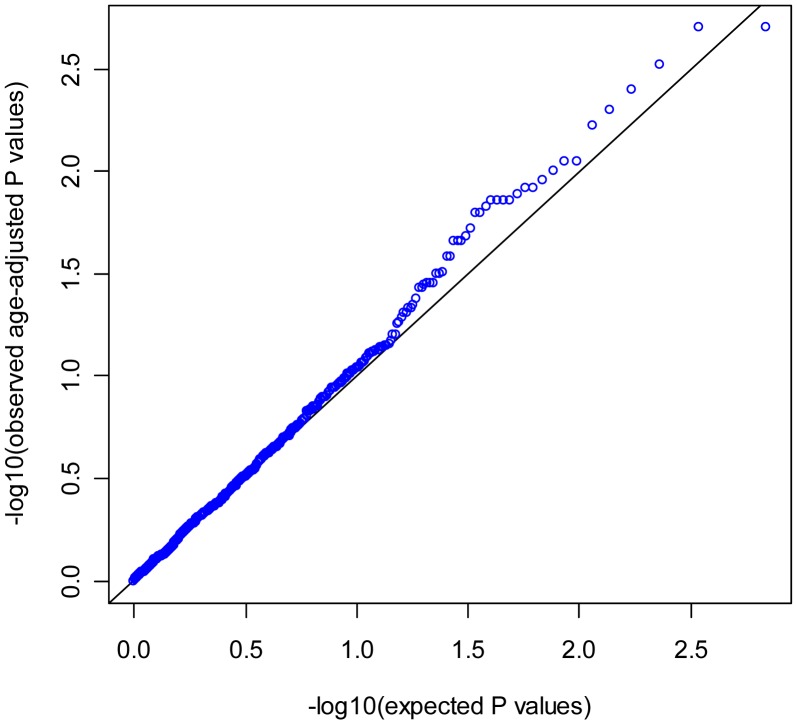
QQ plot for trend tests of 685 ER cofactor SNPs associated with endometrial cancer risk in the Swedish population.

Of the 685 tag SNPs analyzed, nominal P values of less than 0.05 were found for 42 SNPs (6.13%). The Q-Q plot of the observed p-values from the association tests is shown in Figure 1, in which genomic control inflation factor is 1.058, with no clear evidence of deviation from the null. The most significant association was observed at SNP rs130052 (MAF  = 0.13) within the *CREBBP* gene (odds ratio (OR) = 1.41 (95% CI 1.16–1.72), age-adjusted P = 0.002). The ORs with or without age-adjustment were similar. No P-values of individual SNPs could, however, survive the Bonferroni correction for multiple testing.

We further evaluated the association of the genetic variants within each individual ER cofactor gene by carrying out multiple variant association analysis through the Admixture Maximum Likelihood (AML) test, to provide a gene-based P value (Table S3). We found the associations of four genes, *CREBBP* (P = 0.017), *NEDD4* (P = 0.030), *NCOA2* (P = 0.037) and *NR0B1* (P = 0.031) with nominal P values <0.05 (Table 2). However, after correction for multiple testing, none of the associations remained significantly associated with endometrial cancer risk.

**Table 2:** Top four most significant genes associated with endometrial cancer risk in the Swedish population.

Function	Gene(alias)	Chr	SNP#	P-value+	Most significant SNP ID	Most significant SNP’s P-value
Coactivator	*CREBBP*	16	18	0.017	rs130052	0.00046
Coactivator	*NEDD4*	15	34	0.03	rs8033275	0.0066
Coactivator	*NCOA2*	8	27	0.037	rs10216778	0.0051
Corepressor	*NROB1*	X	9	0.031	rs1034948	0.012

+ P value is based on 5000 permutations in AML test for the specific gene.

In view of ER cofactor genes playing their roles via protein complexes, we investigated the associations of the five ER cofactor complexes using AML, namely the P160 family related histone acetylation & methylation complexes (containing *CARM1*, *PRMT1*, *CREBBP*, *EP300*, *NCOA1*, *NCOA2* and *NCOA3*); the RNA processing complex (*PPARGC1A*, *PPARGC1B* and *SRA*); the Pol II recruitment complex (*MED13* and *PBP*); the ligand-dependent corepressors (*NRIP1* and *LCoR*) and the histone deacetylases complex (*HDAC7* and *NCoR1*). Among the five complexes, the P160 family related histone acetylation and methylation complex showed association (P_overall_ = 0.023), but the association could not survive the correction for testing five independent complex groups (P_overall_ = 0.115) (Table 3).

**Table 3:** Global P values of AML tests for genetic association between the tag SNPs in the ER cofactor complexes and endometrial cancer risk in the Swedish population.

Complex	Gene	#tagSNPs	P_global_
Histone acetylation & methylation	*CARM1*	4	0.023
	*PRMT1*	3	
	*NCOA1*	20	
	*NCOA2*	27	
	*NCOA3*	18	
	*CREBBP*	18	
	*EP300*	10	
RNA processing	*PPARGC1A*	46	0.674
	*PPARGC1B*	41	
	*SRA1*	5	
Pol II recruitment	*MED13*	6	0.318
	*PBP*	4	
Ligand-dependent corepressors	*NRIP1*	9	0.586
	*LCoR*	5	
Histone deacetylase	*HDAC7*	10	0.576
	*NCoR1*	3	

P-values were based on 5000 permutations.

As the three individual P160 histone acetylation & methylation complexes (*NCOA1*, *NCOA2* and *NCOA3*) play a distinct role in the transcriptional regulation, we further investigated the associations of these sub-complexes using the AML test. While the *NCOA1* and *NCOA3* sub-complexes did not show association, the *NCOA2* sub-complex demonstrated a significant association (P_overall_ = 0.011) (Table 4), which remained significant after correction for testing three sub-complexes (P_overall_ = 0.033).

**Table 4:** Sub-complex AML tests for genetic association between the tag SNPs in the histone acetylation & methylation and endometrial cancer risk in the Swedish population.

Sub-complex	Gene	#tagSNPs	P_global_
*NCOA1* complex	*CARM1*	4	0.064
	*PRMT1*	3	
	*NCOA1*	20	
	*CREBBP*	18	
	*EP300*	10	
*NCOA2* complex	*CARM1*	4	0.011
	*PRMT1*	3	
	*NCOA2*	27	
	*CREBBP*	18	
	*EP300*	10	
*NCOA3* complex	*CARM1*	4	0.067
	*PRMT1*	3	
	*NCOA3*	18	
	*CREBBP*	18	
	*EP300*	10	

### Validation Analysis Using an in Silico Replication Set

Although the cumulative genetic variants in the P160 family complex were not significant after multiple adjustments, the further sub*-*complex analysis may indicate the source of signal located on *NCOA2* sub-complex. Therefore, we attempted to validate the result in an independent European dataset of 1265 endometrial cancer cases and 5190 controls for which GWAS genotyping data exists [19]. We extracted SNPs from the Illumina Infinium 610K array panel located within 5 kb flanking the five genes of the sub-complex. In total, 65 SNPs were identified and 53 SNPs passed quality control (Table S4). The coverage of common SNPs was 96% in the *NCOA2*, 86% in the *CREBBP*, 80% in the *CARM1*, 50% in the *EP300* and 20% in the *PRMT1* (Table S5). The AML test was applied on each of the five genes and the whole sub-complex (Table 5), but none of them showed significant association (P = 0.71 for the sub-complex).

**Table 5:** Sub-complex AML test for genetic association between the tag SNPs in the NCOA2 complex and endometrial cancer risk in the validation analysis.

Sub-complex	Gene	#tagSNPs	Gene- based P_global_	Complex-based P_global_
*NCOA2* complex	*CARM1*	4	0.116	0.707
	*PRMT1*	3	0.516	
	*NCOA2*	26	0.822	
	*CREBBP*	15	0.469	
	*EP300*	5	0.373	

The sufficient genotyped SNPs and high coverage allowed us to perform imputation analysis on the two genes, *NCOA2* and *CREBBP* in both the Swedish and the European samples. A total of 19 SNPs on *CREBBP* and 270 SNPs on *NCOA2* were shared between the two datasets. We performed meta-analysis of the results for these SNPs across the two datasets. However, no SNP was found to be significantly associated with the risk of endometrial cancer (Table 6 and Table S6).

**Table 6:** List of top five SNPs in the combined analysis in the NCOA2 and CREBBP gene respectively.

Gene	Chr	SNP	BP	OR(95%CI)_ Swedish	OR(95%CI)_ GWAS	OR(95%CI)_ meta-analysis	P_ meta-analysis
*NCOA2*	8	rs1531362	71315749	0.86(0.64,1.16)+	0.89(0.76,1.05+	0.89(0.77,1.02)	0.1
*NCOA2*	8	rs7818867	71286488	0.83(0.66,1.04)	0.94(0.82,1.08)	0.91(0.81,1.02)	0.11
*NCOA2*	8	rs17675762	71272507	0.86(0.68,1.07)+	0.93(0.81,1.07)	0.91(0.81,1.02)	0.12
*NCOA2*	8	8-71359643	71359643	1.38(1.07,1.79)	1.02(0.86,1.21)	1.12(0.97,1.29)	0.12
*NCOA2*	8	rs11777228	71407844	1.36(0.97,1.89)+	1.08(0.85,1.37)	1.16(0.96,1.42)	0.13
*CREBBP*	16	rs12599143	3790417	1.42(1.15,1.76)	0.99(0.86,1.15)	1.11(0.99,1.26)	0.079
*CREBBP*	16	rs130005	3768349	1.45(1.18,1.77)	0.97(0.83,1.13)	1.12(0.99,1.26)	0.083
*CREBBP*	16	rs129963	3736148	1.15(1.0,1.32)+	1.04(0.95,1.13)+	1.07(0.99,1.15)	0.085
*CREBBP*	16	rs2239317	3860741	1.37(1.12,1.68)+	0.97(0.84,1.12)	1.09(0.97,1.23)	0.16
*CREBBP*	16	rs11644593	3811287	1.40(1.13,1.75)	0.97(0.83,1.13)	1.09(0.96,1.24)	0.18

+ Real genotyped SNPs.

## Discussion

To our knowledge, this is the first comprehensive analysis of the association between the polymorphisms of ER cofactor genes and endometrial cancer risk. We did not find significant association between single SNPs or individual genes associated with endometrial cancer risk. Although the genetic variation of the *NCOA2* complex as a whole is marginally associated with endometrial cancer risk in the Swedish population, we failed to validate this finding in an independent study of subjects of European ancestry.

Perissi and Rosenfeld [13] described a vast number of coregulator-complexes that are engaged in ER mediated transcriptional regulation with various functions and enzymatic activities. A study conducted by Lee and colleagues demonstrated that *NCOA2*, *CARM1* and *EP300* or *CREBBP* form a ternary complex that could enhance estrogen receptor (ER) function in a synergistic manner [15]. Although the discovery analysis in our study demonstrated with marginal significance that the cumulative effects of multiple variants of *NCOA2* complex may have a contribution to the risk of endometrial cancer, the association failed to be replicated in an independent sample.

It has been reported [20], [21] that ER coactivators are more commonly expressed in ER-positive endometrial cancer or well-differentiated, hormone-related endometrial cancer rather than ER-negative endometrial cancer. It is therefore likely that the ER cofactors regulate estrogen binding to the estrogen receptors in ER-positive endometrial cancer. Unfortunately, we were unable to perform the sub-group analysis due to sample size limitation and ER status information deficiency. As *NCOA2* complex is formed by 5 genes (*NCOA2*, *CARM1*, *CREBBP*, *PRMT1* and *EP300)* and the coverage of common SNPs is low (50% in *EP300* and 20% in *PRMT1*) in the validation samples, we were not able to impute the SNPs analyzed in the discovery study. Age and other risk factors could potentially affect the results, but unfortunately, we could not perform adjustment due to lack of information in the validation study.

Population stratification is an important issue for genetic association study. For the validation data of this study, all the controls were from UK and had been carefully examined to exclude any subjects of non-European ancestry [22]. All the cases were from Australian and were also of European ancestry. Therefore, the controls and cases are of the same ethnic ancestry. Furthermore, this dataset has been used in a previously published GWAS analysis [19] where PCA analysis showed that population stratification is negligible. Consistently, the λ_GC_ value of the genome-wide results is very small (1.04). Taken together, these results suggested that the cases and the controls of the validation dataset used in the current study were well matched genetically, and the genetic association results of the current study should not suffer the adverse impact of population stratification.

The current study provided a comprehensive analysis of common genetic variants within the ER cofactor genes. First, the large number of tag SNPs covered an average of 91% of common variation (MAF>5%) within the 60 candidate genes based on HapMap CEU data (NCBI36) (Table S1). The average coverage decreased to 64% based on the 1000Genomes CEU data (NCBI37), but the number of captured SNPs increased from 2585 to 5084. Imputation analysis was performed in the discovery and validation samples to evaluate additional untyped SNPs and to ensure that the same set of polymorphisms were analyzed in the discovery and replication samples. With a total sample size of 1829 cases and 6700 controls, our study had an overall power of 85% at a significance level of 0.05 to detect a causal allele with MAF over 0.1 and OR over 1.2 for endometrial cancer. However, we have not found any evidence of association, despite the fact that the association was evaluated by using individual SNP, genes or ER cofactor complex based tests. Therefore, our study has demonstrated that the common polymorphisms within these genes are unlikely to play a significant role in overall endometrial cancer risk in European population. However, we could not exclude the possibility that some SNPs could be associated with the endometrial cancer survival. Further studies will be needed to examine if common variants with weaker effect or rare variants within these genes may play a role in influencing endometrial cancer risk and survival.

## Materials and Methods

### Ethics Statement

This study was approved by the Institutional Review Boards in Sweden and the National University of Singapore, the National Health and Medical Research Council of Australia and the Wellcome Trust Case Control Consortium UK. The patients in this manuscript have given written informed consent to publication of their case details.

### Study Population and DNA Extraction

#### Swedish population

Table 1 summarizes the origins, and numbers of cases and controls used in this study. Subjects were from two independent populations from Sweden and the stage 1 sample set of a recently published endometrial cancer GWAS [19]. Details of the Swedish population selection process for this study have been published earlier [23], [24]. In brief, 564 of all endometrial cancer cases among women 50–74 years of age were identified through the nation-wide cancer registries in Sweden between 1994 and 1995. During that period, 1510 age-frequency matched controls were randomly selected from the Swedish Registry of Total Population. All cases and controls without any previous malignancy were recruited into the study. Only women with an intact uterus were considered as eligible controls. All eligible subjects provided risk factor information including age, reproductive history and body mass index (Table S2), which were obtained via questionnaires.

#### *In silico* validation dataset

Our validation data were drawn from the 1st stage of a GWAS of endometrial cancer, detailed elsewhere [19]. In summary, the stage 1 cases were primary endometrioid subtype endometrial cancer patients reporting European ancestry from Australia (the Australian National Endometrial Cancer Study (ANECS)) and the UK (Studies of Epidemiology and Risk factors in Cancer Heredity (SEARCH) Stage 1 controls were genotyped as part of the Wellcome Trust Case Control Consortium (WTCCC) from England, and included 2,694 controls from the 1958 Birth Cohort (1958BC) (a population-based study in the United Kingdom of individuals born in 1 week in 1958) and 2,496 controls identified through the UK National Blood Service (NBS) [22]. Information on epidemiological risk factors of case subjects from ANECS and SEARCH were collected using respective questionnaires from the two studies. However, this information was not available for the present study.

For the discovery and validation samples, bio-specimen was collected using written informed-consent procedures approved by the respective institutional review boards.

### Candidate Gene, Tagging SNP Selection and Genotyping

The details of ER cofactor genes and tag SNP selection have been previously described [25]. In brief, gene selections were based on the criteria that the gene should code for an ER cofactor protein. We chose tag SNPs within the 60 candidate genes based on the HapMap CEU data (Rel #22/phase II Apr07, on NCBI B36 assembly, dbSNP b126). Using a pair-wise SNP tagging approach with r^2^>0.8 in Haploview [26] (Version 4), 806 tagging SNPs with MAF over 0.05 were selected within introns, exons and the 5 Kb region flanking of each gene. Overall 790 tagSNPs across 60 genes were successfully designed and genotyped in all available DNA samples from cases and controls with the Illumina GoldenGate Assay following the manufacturers’ instructions. Among them, 105 SNPs were excluded on the basis of 81 SNPs having a call rate less than 0.96, 6 SNPs failing a Hardy-Weinberg Equilibrium test (P< (0.05/685)) and 18 SNPs having a MAF <0.01. Finally, 685 tagSNPs were included in the statistical analysis. Genotyping was duplicated in 2% of samples and there was concordance of >99% between duplicated samples, suggesting high genotyping accuracy.

For the validation analysis, the SNPs on the gene *NCOA2*, *CREBBP*, *PRMT1*, *EP300* and *CARM1* were drawn from the 610 K panel based on the physical position of 5 kb flank of specific gene regions. The details of genotyping have been described previously [19]. Briefly, cases were genotyped with Illumina Infinium 610 K array and controls were genotyped using an Illumina Infinium 1.2 M array (Table 1). The following criteria were used for SNPs filtering: call rate ≥95% if MAF ≥5% (or call rate ≥99% if MAF <5%), HWE P>10^−12^ (cases) (or HWE P>10^−7^ if no discrepancy with the control groups) and P<10^−6^ (controls). The duplicate concordance was over 99.9%. Cases and controls were restricted to the following criteria: the sex of all samples was confirmed to be female; the identity-by-descent analysis based on identity-by-state was conducted to detect first-degree cryptic relationships; the principle components analysis (PCA) was utilized to remove non-European ancestry and all samples with a low or high heterozygosity (<0.65 or >0.68) or call rate <97% were excluded. A total of 1265 cases and 5190 controls were used for analysis.

### Statistical Analysis

To assess the risk association between SNPs and endometrial cancer risk, per-allele odds ratio (ORs) and 95% confidence intervals were estimated using a logistic regression in both Swedish and validation GWAS genotyping data. The Cochran–Armitage trend test was used to calculate P values. As the controls were younger than cases in the Swedish study, age at diagnosis/enrolment (as a continuous variable) was included for adjustment. We used a Bonferroni correction to adjust for multiple testing (685 tests of association), although this is conservative as not all of the tests are independent. As the information on age was absent from provided GWAS data, we performed an un-adjusted analysis for the validation set.

Overall evidence of association between the genetic variants in the ER cofactor complex and endometrial cancer risk was evaluated using the Admixture Maximum Likelihood (AML) method, which is described in detail in Tyrer et al [27] and in our previous study [18]. Briefly, the software for the AML test assesses the experiment-wise significance by examining the empirical distribution of single marker test statistics based on a “pseudo-likelihood ratio” test, comparing the ratio of values of the optimized likelihoods under the null and alternative hypotheses for the observed data, with the corresponding values obtained from data sets with case-control status permuted randomly. The method is based upon fitting a mixture model to the distribution of the test statistics, and has two components, one representing SNPs which are independent of the case-control status, the other representing SNPs associated with case-control status. In order to determine whether there exists a cumulative effect from multiple variants, the Cochran-Armitage test statistics for the associated SNPs are assumed to all have the same non-centrality parameter value (chi-squared). The common effect size of the associated SNPs within the complex is also estimated through the non-centrality parameter. We performed the AML-based global test of association for the 5 ER cofactor complexes, 3 sub-complexes and 60 ER cofactor genes specific analysis in Swedish analysis, as well as for NCOA2 sub-complex in the validation data analysis. This test assesses the experiment-wise significance by examining the empirical distribution of single marker test statistics, in order to determine whether there exists a cumulative effect from multiple variants.

For the imputation analysis of *NCOA2* (Chr8: 71181–71484 kbp) and *CREBBP* (Chr16: 3720–3875 kbp), we imputed the two regions in the two datasets: 2074 Swedish samples and 6455 validation samples, by using their genotyped SNPs whose genotypes all passed the QC thresholds (call rate >90%, MAF >1%, HWE P>10^−6^ in controls). Imputation was performed by using the data from 1000 Genomes [28] as reference panel 0 and the data from HapMap III [29] as reference panel 1 (CEU data) in a single imputation analysis, as recommended by the authors of Impute2. Imputed genotypes with probability less than 90% were excluded; and SNPs with impute info less than 80%, MAF less than 1%, HWE P<0.0001 in controls and missing rate greater than 10% of genotypes were dropped from further analysis. Association testing was performed in PLINK [29] by using Cochran-Armitage trend test to analyze the genotype–phenotype association in both studies. In the meta-analysis, the Cochran–Mantel–Haenszel test was applied to test genotype-phenotype association in the combined samples by treating the two individual samples as independent studies. The Breslow-Day test and the Q test were performed to evaluate the significance of heterogeneity among individual studies.

SNP association analyses were performed using STATA version 8.0 (StataCorp, College station, TX, USA). Linkage disequilibrium (LD) calculation was performed in Haploview version 4.1 [26]. The AML analysis was performed using a software obtained from the authors of the method [27]. The software Quanto version 1.2.3 was used for power estimation [30]. The software IMPUTE version 2 [31], [32] was used for imputing genotype data of untyped SNPs.

## Supporting Information

Table S1 Coverage evaluation of common variant in 60 ER cofactor genes.
(DOC)

Table S2 Selected characteristics of discovery sample set in Swedish population.
(DOC)

Table S3 Gene-based AML test in endometrial cancer risk in Swedish population.
(DOC)

Table S4 P-values of 53 SNPs extracted from GWAS after PCA adjustment.
(DOC)

Table S5 Coverage comparison of 5 NCOA2 sub-complex genes between Swedish study and GWAS analysis.
(DOC)

Table S6 Shared imputed and genotyped SNPs in both Swedish and GWAS analysis on NCOA2 and CREBBP genes.
(DOC)
