# Supplementary material for: Genetic Variants in ER Cofactor Genes and Endometrial Cancer Risk
Source: PLoS One. 2012 Aug 2;7(8):e42445. doi: 10.1371/journal.pone.0042445 (PMC3411617; doi:10.1371/journal.pone.0042445)
Supplement: Table S1 — Coverage evaluation of common variant in 60 ER cofactor genes. (DOC) [file pone.0042445.s001.doc]

Table S1. Coverage evaluation of common variant in 60 ER cofactor genes

| Gene | Chr | #Tag | Successful genotyped tags # | Captured SNPs #_HapMap_NCBI36 | Total SNPs # _HapMap_NCBI36 | Coverage†_HapMap(NCBI36) | Captured SNPs #_1000Genome(NCBI37) | Total SNPs # _1000Genome(NCBI37) | Coverage†_1000Genome(NCBI37) |
| --- | --- | --- | --- | --- | --- | --- | --- | --- | --- |
| *NR0B2* | 1 | 7 | 5 | 6 | 6 | 100% | 11 | 23 | 48% |
| *Calmodulin2* | 2 | 8 | 6 | 20 | 24 | 83% | 28 | 52 | 54% |
| *FOXO1* | 2 | 11 | 9 | 32 | 34 | 94% | 71 | 96 | 74% |
| *NCOA1* | 2 | 23 | 20 | 106 | 106 | 100% | 183 | 222 | 82% |
| *TAF1B* | 2 | 18 | 15 | 136 | 148 | 91% | 257 | 464 | 55% |
| *SIAH2* | 3 | 4 | 2 | 4 | 6 | 66% | 4 | 11 | 36% |
| *PPARG* | 3 | 35 | 30 | 112 | 112 | 100% | 231 | 267 | 87% |
| *PPARGC1A* | 4 | 55 | 46 | 97 | 100 | 97% | 143 | 214 | 67% |
| *COUP-TF* | 5 | 3 | 3 | 4 | 4 | 100% | 7 | 10 | 70% |
| *NSD1* | 5 | 10 | 8 | 38 | 43 | 88% | 55 | 137 | 40% |
| *PPARGC1B* | 5 | 51 | 41 | 131 | 162 | 80% | 232 | 289 | 80% |
| *SRA1* | 5 | 6 | 5 | 11 | 11 | 100% | 11 | 17 | 65% |
| *NCOA7* | 6 | 38 | 35 | 115 | 115 | 100% | 206 | 238 | 87% |
| *TRIM24* | 7 | 9 | 8 | 47 | 48 | 97% | 98 | 134 | 73% |
| *RPL7* | 8 | 5 | 5 | 7 | 7 | 100% | 13 | 31 | 42% |
| *SHARPIN* | 8 | 3 | 3 | 6 | 6 | 100% | 20 | 25 | 80% |
| *NCOA2* | 8 | 27 | 27 | 140 | 146 | 96% | 356 | 383 | 93% |
| *BAG1* | 9 | 3 | 2 | 7 | 10 | 70% | 13 | 20 | 65% |
| *ARA70* | 10 | 6 | 5 | 10 | 11 | 90% | 23 | 25 | 92% |
| *ASC* | 10 | 7 | 7 | 25 | 25 | 100% | 39 | 66 | 59% |
| *LCoR* | 10 | 5 | 5 | 38 | 38 | 100% | 106 | 114 | 93% |
| *RBM14* | 11 | 3 | 2 | 2 | 3 | 66% | 5 | 12 | 42% |
| *CCND1* | 11 | 5 | 5 | 5 | 6 | 83% | 7 | 23 | 30% |
| *CALCOCO1* | 12 | 10 | 8 | 26 | 28 | 92% | 32 | 58 | 55% |
| *DDX54* | 12 | 3 | 3 | 7 | 8 | 87% | 41 | 49 | 84% |
| *NCOR2* | 12 | 93 | 69 | 131 | 144 | 90% | 235 | 494 | 48% |
| *PHB2* | 12 | 3 | 2 | 1 | 1 | 100% | 3 | 14 | 21% |
| *SMRT* | 12 | 14 | 10 | 12 | 19 | 63% | 18 | 78 | 23% |
| *NR2C1* | 12 | 13 | 11 | 23 | 27 | 85% | 52 | 81 | 64% |
| *Calmodulin1* | 14 | 7 | 4 | 7 | 14 | 50% | 15 | 39 | 38% |
| *CAPER-beta* | 14 | 8 | 7 | 20 | 21 | 95% | 40 | 48 | 83% |
| *ESR2* | 14 | 20 | 20 | 70 | 70 | 100% | 165 | 201 | 82% |
| *MTA1* | 14 | 4 | 4 | 5 | 5 | 100% | 8 | 30 | 27% |
| *SNW1* | 14 | 11 | 8 | 42 | 48 | 87% | 135 | 189 | 71% |
| *NEDD8* | 14 | 5 | 4 | 6 | 8 | 75% | 10 | 22 | 45% |
| *UBE3A* | 15 | 7 | 7 | 48 | 48 | 100% | 116 | 146 | 79% |
| *NEDD4* | 15 | 31 | 34 | 128 | 128 | 100% | 477 | 603 | 79% |
| *CREBBP* | 16 | 21 | 18 | 21 | 22 | 95% | 33 | 61 | 54% |
| *SMARCE1* | 17 | 3 | 3 | 18 | 18 | 100% | 29 | 32 | 91% |
| *BRCA1* | 17 | 7 | 6 | 42 | 46 | 91% | 138 | 151 | 91% |
| *DDX5* | 17 | 7 | 5 | 16 | 16 | 100% | 12 | 22 | 55% |
| *NCoR1* | 17 | 4 | 3 | 57 | 74 | 77% | 155 | 173 | 90% |
| *PELP1* | 17 | 9 | 6 | 18 | 18 | 100% | 48 | 102 | 47% |
| *SUPT6H* | 17 | 4 | 4 | 7 | 7 | 100% | 21 | 26 | 81% |
| *MED13* | 17 | 8 | 6 | 29 | 31 | 93% | 99 | 126 | 79% |
| *SMAD4* | 18 | 3 | 3 | 24 | 24 | 100% | 38 | 41 | 93% |
| *Calmodulin3* | 19 | 6 | 4 | 4 | 6 | 66% | 11 | 35 | 31% |
| *CARM1* | 19 | 6 | 4 | 12 | 15 | 80% | 34 | 56 | 61% |
| *PRMT1* | 19 | 4 | 3 | 4 | 5 | 80% | 8 | 21 | 38% |
| *SAFB* | 19 | 5 | 4 | 14 | 18 | 77% | 26 | 35 | 74% |
| *SAFB2* | 19 | 10 | 9 | 15 | 16 | 93% | 32 | 42 | 76% |
| *NCOA6* | 20 | 5 | 5 | 49 | 49 | 100% | 102 | 142 | 72% |
| *CAPER-alpha* | 20 | 5 | 4 | 36 | 39 | 92% | 63 | 108 | 58% |
| *CEBPB* | 20 | 5 | 4 | 3 | 3 | 100% | 3 | 8 | 38% |
| *NCOA3* | 20 | 20 | 18 | 98 | 98 | 100% | 251 | 302 | 83% |
| *NRIP1* | 21 | 9 | 9 | 17 | 20 | 85% | 22 | 27 | 81% |
| *Tip60* | 21 | 54 | 47 | 229 | 248 | 92% | 363 | 516 | 70% |
| *EP300* | 22 | 10 | 10 | 42 | 42 | 100% | 98 | 115 | 85% |
| *RBFOX2* | 22 | 21 | 16 | 21 | 21 | 100% | 18 | 42 | 43% |
| *NR0B1* | X | 12 | 9 | 9 | 9 | 100% | 14 | 33 | 42% |
| Total |  | 806 | 685 | 2410 | 2585 | 90.77% | 5084 | 7141 | 64% |

Criteria: MAF>=0.05, r^2>0.8, HWE>0.001 (but HWE criteria not applicable to the SNPs on chromosome X)

† Number of captured SNPs divided by total number of SN
