# Supplementary material for: Genetic Variants in ER Cofactor Genes and Endometrial Cancer Risk
Source: PLoS One. 2012 Aug 2;7(8):e42445. doi: 10.1371/journal.pone.0042445 (PMC3411617; doi:10.1371/journal.pone.0042445)
Supplement: Table S2 — Selected characteristics of discovery sample set in Swedish population. (DOC) [file pone.0042445.s002.doc]

Table S2 Selected characteristics of discovery sample set in Swedish population

| **Characteristic** | **Number (case/ctrl)** | **Mean (case/ctrl)** | **P value** |
| --- | --- | --- | --- |
| Age | 564 / 1510 | 63.66 / 62.74 | 0.005 |
| Age at first pregnancy | 457 / 1354 | 24.74 / 24.77 | 0.92 |
| Age at last birth | 487 / 1354 | 29.55 / 30.40 | 0.002 |
| Age at menarche | 564 / 1510 | 13.43 / 13.53 | 0.16 |
| Age at menopause | 490 / 1454 | 51.01 / 50.07 | <0.0001 |
| Parity | 564 / 1510 | 1.93 / 2.14 | 0.001 |
| BMI (recent) | 563 / 1493 | 27.18 / 25.55 | <0.0001 |
