# Supplementary material for: Genetic Variants in ER Cofactor Genes and Endometrial Cancer Risk
Source: PLoS One. 2012 Aug 2;7(8):e42445. doi: 10.1371/journal.pone.0042445 (PMC3411617; doi:10.1371/journal.pone.0042445)
Supplement: Table S3 — Gene-based AML test in endometrial cancer risk in Swedish population. (DOC) [file pone.0042445.s003.doc]

Table S3. Gene-based AML test in endometrial cancer risk in Swedish population.

| Function | Gene | Chr | SNP# | P-value+ |
| --- | --- | --- | --- | --- |
| coactivator | *ARA70* | 10 | 5 | 0.512 |
| coactivator | *TRIP4* | 10 | 7 | 0.997 |
| coactivator | *NCOA6* | 20 | 5 | 0.456 |
| coactivator | *SMARCE1* | 17 | 3 | 0.66 |
| corepressor | *BRCA1* | 17 | 6 | 0.909 |
| coactivator | *Calmodulin1* | 14 | 4 | 0.162 |
| coactivator | *Calmodulin2* | 2 | 6 | 0.667 |
| coactivator | *Calmodulin3* | 19 | 4 | 0.357 |
| coactivator | *RBM39* | 20 | 4 | 0.25 |
| coactivator | *RBM23* | 14 | 7 | 0.877 |
| coactivator | *CARM1* | 19 | 4 | 0.473 |
| coactivator | *CEBPB* | 20 | 4 | 0.638 |
| coactivator | *RBM14* | 11 | 2 | 0.675 |
| coactivator | *CALCOCO1* | 12 | 8 | 0.912 |
| corepressor | *COUP-TF* | 5 | 3 | 0.168 |
| coactivator | *CREBBP* | 16 | 18 | 0.017 |
| coactivator | *CCND1* | 11 | 5 | 0.811 |
| coactivator | *DDX5* | 17 | 5 | 0.358 |
| corepressor | *DDX54* | 12 | 3 | 0.706 |
| coactivator | *UBE3A* | 15 | 7 | 0.517 |
| coactivator | *EP300* | 22 | 10 | 0.595 |
| corepressor | *ESR2* | 14 | 20 | 0.276 |
| corepressor | *FOXO1* | 2 | 9 | 0.629 |
| coactivator | *NEDD4* | 15 | 34 | 0.03 |
| corepressor | *NR2C1* | 12 | 11 | 0.628 |
| coactivator | *RPL7* | 8 | 5 | 0.499 |
| corepressor | *LCOR* | 10 | 5 | 0.397 |
| corepressor | *SIAH2* | 3 | 2 | 0.386 |
| corepressor | *MTA1* | 14 | 4 | 0.274 |
| coactivator | *NCOA1* | 2 | 20 | 0.265 |
| coactivator | *NCOA2* | 8 | 27 | 0.037 |
| coactivator | *NCOA3* | 20 | 18 | 0.294 |
| coactivator | *SNW1* | 14 | 8 | 0.617 |
| coactivator | *NCOA7* | 6 | 35 | 0.67 |
| corepressor | *NCoR1* | 17 | 3 | 0.268 |
| corepressor | *NCOR2* | 12 | 69 | 0.32 |
| corepressor | *GMPR2* | 14 | 4 | 0.96 |
| corepressor | *NR0B1* | X | 9 | 0.031 |
| corepressor | *NROB2* | 1 | 5 | 0.634 |
| corepressor | *NSD1* | 5 | 8 | 0.109 |
| coactivator | *PELP1* | 17 | 6 | 0.439 |
| coactivator | *PPARGC1B* | 5 | 41 | 0.882 |
| coactivator | *PPARG* | 3 | 30 | 0.917 |
| coactivator | *PPARGC1A* | 4 | 46 | 0.257 |
| coactivator | *PRMT1* | 19 | 3 | 0.238 |
| coactivator | *BAG1* | 9 | 2 | 0.299 |
| corepressor | *RBFOX2* | 22 | 16 | 0.817 |
| corepressor | *PHB2* | 12 | 2 | 0.576 |
| corepressor | *NRIP1* | 21 | 9 | 0.602 |
| corepressor | *SAFB* | 19 | 4 | 0.587 |
| corepressor | *SAFB2* | 19 | 9 | 0.983 |
| corepressor | *SHARPIN* | 8 | 3 | 0.978 |
| corepressor | *SMAD4* | 18 | 3 | 0.67 |
| corepressor | *HDAC7* | 12 | 10 | 0.718 |
| coactivator | *SUPT6H* | 17 | 4 | 0.931 |
| coactivator | *SRA1* | 5 | 5 | 0.168 |
| corepressor | *TAF1B* | 2 | 15 | 0.563 |
| coactivator | *TRIM24* | 7 | 8 | 0.685 |
| coactivator | *KAT5* | 21 | 47 | 0.073 |
| coactivator | *MED13* | 17 | 6 | 0.305 |

+: AML test based on 5000 Permutations
