# Supplementary material for: Genetic Variants in ER Cofactor Genes and Endometrial Cancer Risk
Source: PLoS One. 2012 Aug 2;7(8):e42445. doi: 10.1371/journal.pone.0042445 (PMC3411617; doi:10.1371/journal.pone.0042445)
Supplement: Table S4 — P-values of 53 SNPs extracted from GWAS after PCA adjustment. (DOC) [file pone.0042445.s004.doc]

Table S4 P-values of 53 SNPs extracted from GWAS after PCA adjustment.

| SNP | Chr | Position | all_alleles | ctrlMAF | caseMAF | eg_chi | eg_p |
| --- | --- | --- | --- | --- | --- | --- | --- |
| rs4545135 | 8 | 71201269 | AC | 0.245 | 0.246 | 0.029 | 0.865 |
| rs12547963 | 8 | 71202597 | AG | 0.076 | 0.071 | 0.386 | 0.534 |
| rs13260857 | 8 | 71204984 | AC | 0.227 | 0.223 | 0.18 | 0.671 |
| rs3812430 | 8 | 71212926 | CA | 0.122 | 0.119 | 0.357 | 0.55 |
| rs3812429 | 8 | 71264273 | AG | 0.122 | 0.121 | 0.085 | 0.77 |
| rs4623463 | 8 | 71284972 | AG | 0.397 | 0.391 | 0.168 | 0.682 |
| rs10957517 | 8 | 71287989 | GA | 0.071 | 0.067 | 0.157 | 0.692 |
| rs4738080 | 8 | 71292367 | GA | 0.072 | 0.072 | 0.112 | 0.737 |
| rs17676138 | 8 | 71297215 | AC | 0.172 | 0.166 | 0.02 | 0.887 |
| rs16936837 | 8 | 71303986 | AG | 0.049 | 0.051 | 0.726 | 0.394 |
| rs1531362 | 8 | 71315749 | GA | 0.082 | 0.074 | 1.331 | 0.249 |
| rs17676564 | 8 | 71320670 | GA | 0.05 | 0.048 | 0.239 | 0.625 |
| rs1460680 | 8 | 71326322 | AG | 0.051 | 0.054 | 0.021 | 0.884 |
| rs2926707 | 8 | 71327234 | AC | 0.276 | 0.274 | 0.01 | 0.919 |
| rs2958367 | 8 | 71327441 | AG | 0.153 | 0.151 | 0.123 | 0.726 |
| rs2926703 | 8 | 71329548 | AG | 0.153 | 0.151 | 0.092 | 0.762 |
| rs2926702 | 8 | 71330548 | AG | 0.13 | 0.116 | 2.861 | 0.091 |
| rs16936880 | 8 | 71362663 | GA | 0.102 | 0.097 | 0.237 | 0.626 |
| rs13260060 | 8 | 71380914 | GA | 0.101 | 0.097 | 0.222 | 0.637 |
| rs17677919 | 8 | 71391161 | AG | 0.173 | 0.166 | 0.064 | 0.8 |
| rs10504472 | 8 | 71403032 | GA | 0.072 | 0.07 | 0.02 | 0.886 |
| rs4738088 | 8 | 71413558 | AC | 0.051 | 0.054 | 0.007 | 0.934 |
| rs10504473 | 8 | 71422886 | AC | 0.153 | 0.15 | 0.172 | 0.678 |
| rs10504474 | 8 | 71423014 | GA | 0.102 | 0.097 | 0.241 | 0.624 |
| rs6472520 | 8 | 71435869 | AG | 0.225 | 0.222 | 0.213 | 0.644 |
| rs6986140 | 8 | 71441140 | GA | 0.225 | 0.221 | 0.22 | 0.639 |
| rs2191416 | 16 | 3710730 | GA | 0.253 | 0.259 | 0.448 | 0.503 |
| rs11076785 | 16 | 3730187 | CA | 0.328 | 0.337 | 0.779 | 0.378 |
| rs129968 | 16 | 3731262 | GA | 0.353 | 0.366 | 1.614 | 0.204 |
| rs129963 | 16 | 3736148 | GA | 0.434 | 0.443 | 0.772 | 0.38 |
| rs17136507 | 16 | 3740546 | AG | 0.079 | 0.086 | 1.104 | 0.293 |
| rs886528 | 16 | 3751557 | AG | 0.451 | 0.435 | 1.778 | 0.182 |
| rs11076786 | 16 | 3751597 | GA | 0.102 | 0.099 | 0.295 | 0.587 |
| rs130021 | 16 | 3772472 | AG | 0.36 | 0.353 | 0.758 | 0.384 |
| rs130023 | 16 | 3773307 | AG | 0.035 | 0.04 | 1.977 | 0.16 |
| rs8046065 | 16 | 3778299 | GA | 0.126 | 0.128 | 0.015 | 0.901 |
| rs3789033 | 16 | 3779536 | AG | 0.235 | 0.23 | 0.742 | 0.389 |
| rs11076787 | 16 | 3792777 | GA | 0.218 | 0.23 | 3.194 | 0.074 |
| rs1296720 | 16 | 3813643 | AC | 0.209 | 0.223 | 2.982 | 0.084 |
| rs2239316 | 16 | 3853996 | AG | 0.252 | 0.244 | 1.431 | 0.232 |
| rs2239318 | 16 | 3864938 | GA | 0.104 | 0.102 | 0.248 | 0.619 |
| rs12460421 | 19 | 10842352 | AG | 0.425 | 0.449 | 4.781 | 0.029 |
| rs1541596 | 19 | 10848013 | GA | 0.483 | 0.472 | 0.919 | 0.338 |
| rs1549926 | 19 | 10863862 | AC | 0.295 | 0.31 | 2.452 | 0.117 |
| rs1529711 | 19 | 10884434 | GA | 0.159 | 0.159 | 0.018 | 0.893 |
| rs3745469 | 19 | 54868983 | GA | 0.097 | 0.093 | 0.704 | 0.401 |
| rs10415880 | 19 | 54880968 | GA | 0.331 | 0.319 | 1.185 | 0.276 |
| rs892149 | 19 | 54886657 | CA | 0.055 | 0.057 | 0.004 | 0.948 |
| rs5758223 | 22 | 39819866 | AG | 0.278 | 0.262 | 1.758 | 0.185 |
| rs4822006 | 22 | 39849308 | GA | 0.367 | 0.354 | 1.126 | 0.289 |
| rs2294976 | 22 | 39894654 | CA | 0.088 | 0.09 | 0.022 | 0.882 |
| rs5758251 | 22 | 39900691 | AG | 0.348 | 0.345 | 0.22 | 0.639 |
| rs1046088 | 22 | 39904329 | AC | 0.034 | 0.038 | 1.276 | 0.259 |
