# Supplementary material for: Genetic Variants in ER Cofactor Genes and Endometrial Cancer Risk
Source: PLoS One. 2012 Aug 2;7(8):e42445. doi: 10.1371/journal.pone.0042445 (PMC3411617; doi:10.1371/journal.pone.0042445)
Supplement: Table S5 — Coverage comparison of 5 NCOA2 sub-complex genes between Swedish study and GWAS analysis. (DOC) [file pone.0042445.s005.doc]

Table S5. Coverage comparison of 5 NCOA2 sub-complex genes between Swedish study and GWAS analysis

| Gene |  | Chr | Start position† | End position† | Successful genotyped tags # | Captured SNPs # (r^2 >0.8) | Total SNPs # | Coverage* |
| --- | --- | --- | --- | --- | --- | --- | --- | --- |
| *CARM1* | Swedish | 19 | 10843253 | 10894447 | 4 | 12 | 15 | 80% |
|  | GWAS |  |  |  | 4 | 12 | 15 | 80% |
| *CREBBP* | Swedish | 16 | 3715057 | 3870122 | 18 | 21 | 22 | 95% |
|  | GWAS |  |  |  | 15 | 19 | 22 | 86% |
| *EP300* | Swedish | 22 | 39818553 | 39906024 | 10 | 42 | 42 | 100% |
|  | GWAS |  |  |  | 5 | 21 | 42 | 50% |
| *NCOA2* | Swedish | 8 | 71186821 | 71478574 | 27 | 140 | 146 | 96% |
|  | GWAS |  |  |  | 26 | 132 | 146 | 90% |
| *PRMT1* | Swedish | 19 | 54872355 | 54883516 | 3 | 4 | 5 | 80% |
|  | GWAS |  |  |  | 3 | 1 | 5 | 20% |

Criteria: MAF>=0.05

† NCBI36, db126

* Number of captured SNPs divide total SNPs number
