# Supplementary material for: Genetic Variants in ER Cofactor Genes and Endometrial Cancer Risk
Source: PLoS One. 2012 Aug 2;7(8):e42445. doi: 10.1371/journal.pone.0042445 (PMC3411617; doi:10.1371/journal.pone.0042445)
Supplement: Table S6 — Shared imputed and genotyped SNPs in both Swedish and GWAS analysis on NCOA2 and CREBBP genes. (DOC) [file pone.0042445.s006.doc]

Table S6 Shared imputed and genotyped SNPs in both Swedish and GWAS analysis on NCOA2 and CREBBP genes.

| Chr | SNP | BP | OR(95%CI)_Swedish | OR(95%CI)_GWAS | OR(95%CI)_meta-analysis | P_meta-analysis |
| --- | --- | --- | --- | --- | --- | --- |
| 8 | rs3892265 | 71174466 | 0.66(0.46,0.96) | 1.03(0.84,1.25) | 0.92(0.78,1.1) | 0.38 |
| 8 | rs3088092 | 71187470 | 0.96(0.75,1.25) | 0.95(0.8,1.13) | 0.95(0.83,1.1) | 0.52 |
| 8 | rs1046013 | 71187836 | 0.65(0.45,0.94) | 1.03(0.84,1.25) | 0.92(0.77,1.09) | 0.33 |
| 8 | rs4546698 | 71192186 | 1.08(0.89,1.3) | 1.03(0.91,1.17) | 1.05(0.94,1.16) | 0.39 |
| 8 | rs4571760 | 71192308 | 1.08(0.89,1.3) | 1.03(0.91,1.17) | 1.05(0.94,1.16) | 0.39 |
| 8 | rs11995219 | 71194751 | 1.08(0.89,1.3) | 1.03(0.91,1.17) | 1.05(0.94,1.16) | 0.4 |
| 8 | rs3763523 | 71196023 | 1.08(0.9,1.31) | 1(0.88,1.14) | 1.03(0.92,1.14) | 0.63 |
| 8 | 8-71197236 | 71197236 | 0.69(0.48,1) | 1.03(0.84,1.25) | 0.93(0.78,1.11) | 0.43 |
| 8 | rs4236980 | 71198169 | 0.98(0.79,1.21) | 1(0.87,1.15) | 0.99(0.88,1.11) | 0.87 |
| 8 | rs3736661 | 71199685 | 0.69(0.48,1) | 1.03(0.84,1.25) | 0.93(0.78,1.11) | 0.43 |
| 8 | rs4545135 | 71201269 | **1.04(0.89,1.22)** | **1(0.91,1.11)** | 1.01(0.93,1.1) | 0.73 |
| 8 | rs2228591 | 71201672 | 0.69(0.48,1) | 1.03(0.84,1.25) | 0.93(0.78,1.11) | 0.43 |
| 8 | rs12547963 | 71202597 | 0.97(0.75,1.26) | **0.93(0.79,1.1)** | 0.94(0.82,1.08) | 0.4 |
| 8 | 8-71203783 | 71203783 | 0.69(0.48,1) | 1.04(0.85,1.26) | 0.94(0.79,1.12) | 0.47 |
| 8 | rs16936746 | 71204780 | 0.68(0.47,0.98) | 1.04(0.85,1.26) | 0.94(0.79,1.11) | 0.45 |
| 8 | 8-71204926 | 71204926 | 0.96(0.74,1.24) | 0.95(0.8,1.13) | 0.96(0.83,1.1) | 0.54 |
| 8 | rs13260857 | 71204984 | **1.02(0.87,1.19)** | **0.98(0.88,1.09)** | 0.99(0.91,1.08) | 0.82 |
| 8 | rs16936749 | 71208737 | 1.33(1.07,1.64) | 0.96(0.83,1.12) | 1.07(0.94,1.21) | 0.31 |
| 8 | rs4074358 | 71208965 | 1.3(1.04,1.64) | 1.01(0.87,1.18) | 1.09(0.96,1.24) | 0.17 |
| 8 | rs3812430 | 71212926 | 0.97(0.78,1.2) | **0.97(0.85,1.11)** | 0.97(0.87,1.09) | 0.63 |
| 8 | rs16936761 | 71214404 | 1.32(1.06,1.65) | 0.96(0.82,1.12) | 1.06(0.93,1.2) | 0.37 |
| 8 | rs12674920 | 71216461 | **1.22(0.95,1.56)** | 0.92(0.76,1.11) | 1.01(0.87,1.18) | 0.86 |
| 8 | rs34726915 | 71218164 | 1.32(1.06,1.65) | 0.96(0.82,1.12) | 1.06(0.93,1.2) | 0.37 |
| 8 | rs4738072 | 71222561 | 0.69(0.48,0.99) | 1.06(0.87,1.29) | 0.95(0.8,1.13) | 0.58 |
| 8 | rs16936768 | 71227757 | 0.67(0.47,0.97) | 1.05(0.86,1.28) | 0.94(0.79,1.12) | 0.52 |
| 8 | 8-71229746 | 71229746 | 0.95(0.73,1.24) | 0.95(0.8,1.13) | 0.95(0.82,1.1) | 0.47 |
| 8 | rs2979638 | 71229922 | 1.32(1.06,1.65) | 0.96(0.82,1.12) | 1.06(0.93,1.2) | 0.37 |
| 8 | rs11786991 | 71230737 | 0.97(0.75,1.26) | 0.95(0.8,1.13) | 0.96(0.83,1.11) | 0.56 |
| 8 | rs2957094 | 71233081 | 1.09(0.9,1.31) | 1(0.88,1.13) | 1.02(0.92,1.13) | 0.67 |
| 8 | rs2977983 | 71233342 | 1.26(0.95,1.65) | 0.91(0.76,1.1) | 1.01(0.86,1.18) | 0.93 |
| 8 | 8-71233649 | 71233649 | 0.95(0.73,1.24) | 0.95(0.8,1.13) | 0.95(0.82,1.1) | 0.47 |
| 8 | rs726285 | 71233741 | 1.32(1.06,1.65) | 0.96(0.82,1.12) | 1.06(0.93,1.2) | 0.37 |
| 8 | rs2272670 | 71234249 | 1.03(0.87,1.21) | 0.97(0.87,1.07) | 0.98(0.9,1.08) | 0.72 |
| 8 | rs2977984 | 71236226 | 1.31(1.05,1.65) | 1(0.86,1.17) | 1.09(0.96,1.24) | 0.19 |
| 8 | rs41391448 | 71236254 | 0.66(0.46,0.95) | 1.02(0.84,1.25) | 0.92(0.77,1.09) | 0.34 |
| 8 | rs2979633 | 71237118 | 1.09(0.9,1.31) | 1(0.88,1.13) | 1.02(0.92,1.13) | 0.67 |
| 8 | rs4236981 | 71238746 | 0.69(0.48,0.99) | 1.06(0.87,1.29) | 0.95(0.8,1.13) | 0.58 |
| 8 | rs2958371 | 71239749 | 1.31(1.04,1.64) | 1(0.86,1.17) | 1.09(0.96,1.23) | 0.2 |
| 8 | rs877181 | 71239842 | 1.09(0.9,1.31) | 1(0.88,1.13) | 1.02(0.92,1.13) | 0.67 |
| 8 | rs3793365 | 71240569 | 0.96(0.74,1.25) | 0.95(0.8,1.13) | 0.95(0.83,1.1) | 0.53 |
| 8 | 8-71242691 | 71242691 | 0.95(0.73,1.24) | 0.95(0.79,1.13) | 0.95(0.82,1.1) | 0.46 |
| 8 | rs4738074 | 71243256 | 0.69(0.48,0.99) | 1.06(0.87,1.29) | 0.95(0.8,1.13) | 0.58 |
| 8 | rs10504468 | 71243580 | 0.69(0.48,0.99) | 1.06(0.87,1.29) | 0.95(0.8,1.13) | 0.58 |
| 8 | rs2958370 | 71245272 | 1.32(1.05,1.65) | 0.99(0.85,1.16) | 1.08(0.95,1.23) | 0.23 |
| 8 | rs7017499 | 71246007 | 1.06(0.87,1.29) | 0.99(0.87,1.12) | 1.01(0.91,1.12) | 0.86 |
| 8 | rs16936790 | 71249022 | 1.08(0.89,1.31) | 1(0.88,1.13) | 1.02(0.92,1.13) | 0.7 |
| 8 | rs4738075 | 71249849 | 1.05(0.89,1.24) | 0.97(0.87,1.08) | 0.99(0.91,1.09) | 0.9 |
| 8 | rs9298156 | 71251102 | 1.08(0.89,1.31) | 1(0.88,1.13) | 1.02(0.92,1.13) | 0.7 |
| 8 | rs9886465 | 71251284 | 1.03(0.87,1.21) | 0.97(0.87,1.08) | 0.99(0.9,1.08) | 0.74 |
| 8 | rs10504469 | 71251571 | 1.3(1.04,1.63) | 0.97(0.83,1.13) | 1.06(0.94,1.2) | 0.36 |
| 8 | rs7003804 | 71252185 | 1.06(0.87,1.29) | 0.98(0.87,1.12) | 1(0.9,1.12) | 0.94 |
| 8 | rs10504470 | 71253000 | 0.97(0.75,1.27) | 0.95(0.8,1.13) | 0.96(0.83,1.1) | 0.54 |
| 8 | 8-71253891 | 71253891 | 0.66(0.45,0.95) | 1.04(0.85,1.27) | 0.93(0.78,1.11) | 0.41 |
| 8 | rs16936806 | 71254006 | 0.68(0.47,0.98) | 1.05(0.86,1.27) | 0.94(0.79,1.12) | 0.5 |
| 8 | rs6995481 | 71254954 | **0.65(0.46,0.93)** | 0.98(0.81,1.2) | 0.89(0.75,1.05) | 0.17 |
| 8 | 8-71255572 | 71255572 | 0.68(0.47,0.97) | 1.05(0.86,1.27) | 0.94(0.79,1.12) | 0.48 |
| 8 | rs7001409 | 71255994 | 1.31(1.05,1.63) | 0.96(0.83,1.12) | 1.06(0.93,1.2) | 0.37 |
| 8 | rs11993276 | 71257913 | 1.3(1.04,1.63) | 0.95(0.82,1.11) | 1.05(0.93,1.19) | 0.44 |
| 8 | rs16936812 | 71258296 | 0.65(0.45,0.93) | 1.02(0.84,1.25) | 0.91(0.77,1.09) | 0.3 |
| 8 | rs16936814 | 71258408 | 0.64(0.44,0.93) | 1.03(0.84,1.25) | 0.92(0.77,1.09) | 0.33 |
| 8 | rs34507548 | 71258906 | 0.68(0.47,0.97) | 1.04(0.86,1.27) | 0.94(0.79,1.12) | 0.47 |
| 8 | 8-71259424 | 71259424 | 0.68(0.47,0.97) | 1.04(0.86,1.27) | 0.94(0.79,1.12) | 0.47 |
| 8 | rs6472512 | 71259595 | 1.31(1.05,1.63) | 0.96(0.83,1.12) | 1.06(0.94,1.2) | 0.36 |
| 8 | rs6998609 | 71260272 | 1.04(0.88,1.23) | 0.98(0.88,1.09) | 1(0.91,1.09) | 0.93 |
| 8 | rs6980582 | 71260318 | 0.68(0.47,0.97) | 1.04(0.86,1.27) | 0.94(0.79,1.12) | 0.47 |
| 8 | rs4512408 | 71261648 | 1.31(1.05,1.64) | 0.96(0.83,1.12) | 1.06(0.94,1.2) | 0.35 |
| 8 | 8-71262450 | 71262450 | 0.67(0.47,0.97) | 1.04(0.86,1.27) | 0.94(0.79,1.11) | 0.45 |
| 8 | rs4738076 | 71263188 | 1.09(0.9,1.32) | 1(0.88,1.13) | 1.02(0.92,1.13) | 0.69 |
| 8 | 8-71263226 | 71263226 | 0.67(0.47,0.97) | 1.04(0.86,1.27) | 0.94(0.79,1.11) | 0.45 |
| 8 | rs7015854 | 71263910 | 1.05(0.85,1.3) | 1(0.87,1.15) | 1.02(0.91,1.14) | 0.78 |
| 8 | rs3812429 | 71264273 | **0.83(0.67,1.03)** | **0.99(0.87,1.13)** | 0.94(0.84,1.05) | 0.3 |
| 8 | rs16936816 | 71265290 | 0.66(0.46,0.95) | 1.04(0.86,1.27) | 0.93(0.78,1.11) | 0.42 |
| 8 | 8-71267258 | 71267258 | 0.64(0.45,0.93) | 1.04(0.86,1.27) | 0.93(0.78,1.1) | 0.38 |
| 8 | rs10087049 | 71268858 | 0.95(0.83,1.1) | 0.95(0.87,1.05) | 0.95(0.88,1.03) | 0.25 |
| 8 | rs4512409 | 71269259 | **0.96(0.83,1.11)** | 0.95(0.87,1.04) | 0.95(0.88,1.03) | 0.24 |
| 8 | rs17675762 | 71272507 | **0.86(0.68,1.07)** | 0.93(0.81,1.07) | 0.91(0.81,1.02) | 0.12 |
| 8 | rs4645588 | 71276152 | 1.33(1.07,1.66) | 0.97(0.83,1.13) | 1.07(0.94,1.21) | 0.31 |
| 8 | 8-71276440 | 71276440 | 0.65(0.45,0.94) | 1.03(0.84,1.25) | 0.92(0.77,1.09) | 0.33 |
| 8 | rs62530460 | 71277349 | 1.05(0.89,1.24) | 0.97(0.87,1.09) | 1(0.91,1.09) | 0.92 |
| 8 | rs4236982 | 71279477 | 1.33(1.07,1.66) | 0.96(0.83,1.12) | 1.07(0.94,1.21) | 0.31 |
| 8 | rs10957516 | 71281605 | **1.15(0.99,1.33)** | 1(0.91,1.11) | 1.04(0.96,1.13) | 0.3 |
| 8 | rs4737302 | 71282831 | 1.38(1.07,1.78) | 0.99(0.87,1.12) | 1.05(0.94,1.18) | 0.38 |
| 8 | rs11775170 | 71283697 | 1.04(0.88,1.23) | 0.99(0.9,1.1) | 1(0.92,1.1) | 0.9 |
| 8 | 8-71285195 | 71285195 | 0.65(0.45,0.94) | 1.03(0.84,1.25) | 0.92(0.77,1.09) | 0.34 |
| 8 | rs7818867 | 71286488 | 0.83(0.66,1.04) | 0.94(0.82,1.08) | 0.91(0.81,1.02) | 0.11 |
| 8 | rs10957517 | 71287989 | 0.96(0.74,1.26) | **0.95(0.8,1.13)** | 0.95(0.82,1.1) | 0.5 |
| 8 | rs4484728 | 71290505 | 1.07(0.88,1.29) | 0.99(0.87,1.12) | 1.01(0.91,1.12) | 0.84 |
| 8 | rs4738079 | 71292241 | **1.27(1.04,1.57)** | 1.01(0.89,1.14) | 1.07(0.96,1.2) | 0.2 |
| 8 | rs4738080 | 71292367 | **1.36(1.07,1.74)** | **1.01(0.85,1.19)** | 1.11(0.96,1.27) | 0.15 |
| 8 | rs4738081 | 71292380 | 0.64(0.44,0.92) | 1.03(0.84,1.25) | 0.91(0.77,1.09) | 0.3 |
| 8 | 8-71293583 | 71293583 | 0.64(0.44,0.92) | 1.03(0.84,1.25) | 0.91(0.77,1.09) | 0.3 |
| 8 | rs4146466 | 71293930 | 1.38(1.06,1.78) | 0.97(0.83,1.13) | 1.05(0.93,1.2) | 0.42 |
| 8 | rs4236983 | 71294904 | 0.64(0.44,0.92) | 1.03(0.84,1.25) | 0.91(0.77,1.09) | 0.3 |
| 8 | 8-71295274 | 71295274 | 0.96(0.73,1.25) | 0.96(0.81,1.14) | 0.96(0.83,1.11) | 0.56 |
| 8 | 8-71296345 | 71296345 | 0.64(0.44,0.92) | 1.03(0.84,1.25) | 0.91(0.77,1.09) | 0.3 |
| 8 | rs11785897 | 71296992 | 1.04(0.84,1.29) | 0.99(0.88,1.13) | 1(0.9,1.12) | 0.92 |
| 8 | rs17676138 | 71297215 | **0.97(0.8,1.18)** | **0.96(0.85,1.08)** | 0.96(0.87,1.06) | 0.45 |
| 8 | rs10086357 | 71298389 | 1.04(0.84,1.29) | 0.99(0.87,1.12) | 1(0.9,1.12) | 0.94 |
| 8 | rs4236984 | 71301906 | 1.04(0.83,1.29) | 0.99(0.87,1.12) | 1(0.9,1.12) | 0.99 |
| 8 | 8-71302490 | 71302490 | 0.96(0.73,1.25) | 0.96(0.8,1.14) | 0.96(0.83,1.11) | 0.56 |
| 8 | rs2977985 | 71305268 | 1.04(0.84,1.3) | 1(0.88,1.13) | 1.01(0.91,1.13) | 0.84 |
| 8 | 8-71305512 | 71305512 | 0.96(0.73,1.25) | 0.96(0.8,1.14) | 0.96(0.83,1.11) | 0.56 |
| 8 | rs2977986 | 71306110 | 1.07(0.88,1.29) | 1(0.88,1.14) | 1.02(0.92,1.13) | 0.7 |
| 8 | rs55812701 | 71306638 | 0.96(0.73,1.25) | 0.96(0.8,1.14) | 0.96(0.83,1.11) | 0.56 |
| 8 | 8-71306985 | 71306985 | 0.64(0.44,0.92) | 1.03(0.84,1.25) | 0.91(0.77,1.09) | 0.3 |
| 8 | rs2926719 | 71308243 | 1.01(0.85,1.19) | 0.97(0.87,1.08) | 0.98(0.89,1.07) | 0.63 |
| 8 | rs2977988 | 71308604 | 1.05(0.87,1.28) | 0.98(0.86,1.11) | 1(0.9,1.11) | 0.99 |
| 8 | rs4737303 | 71309207 | 0.63(0.44,0.91) | 1.03(0.84,1.25) | 0.91(0.76,1.08) | 0.28 |
| 8 | rs2926718 | 71309318 | 1.31(1.05,1.64) | 0.99(0.85,1.15) | 1.08(0.96,1.23) | 0.21 |
| 8 | 8-71311861 | 71311861 | 0.64(0.44,0.92) | 1.03(0.84,1.25) | 0.91(0.77,1.09) | 0.3 |
| 8 | rs2926715 | 71313751 | 1.04(0.85,1.27) | 0.98(0.87,1.11) | 1(0.9,1.11) | 0.95 |
| 8 | rs2926714 | 71314054 | 1.38(1.07,1.79) | 0.98(0.84,1.14) | 1.07(0.94,1.22) | 0.33 |
| 8 | 8-71315305 | 71315305 | 0.64(0.44,0.92) | 1.04(0.86,1.27) | 0.92(0.78,1.1) | 0.36 |
| 8 | rs1531362 | 71315749 | **0.86(0.64,1.16)** | **0.89(0.76,1.05)** | 0.89(0.77,1.02) | 0.1 |
| 8 | 8-71319693 | 71319693 | 0.63(0.44,0.91) | 1.05(0.86,1.28) | 0.92(0.78,1.1) | 0.37 |
| 8 | rs2958366 | 71320650 | 1.07(0.88,1.3) | 1(0.89,1.14) | 1.02(0.92,1.14) | 0.65 |
| 8 | rs17676564 | 71320670 | **0.99(0.76,1.3)** | **0.96(0.78,1.17)** | 0.97(0.82,1.14) | 0.71 |
| 8 | 8-71323226 | 71323226 | 0.63(0.44,0.91) | 1.05(0.86,1.28) | 0.93(0.78,1.1) | 0.38 |
| 8 | 8-71323345 | 71323345 | 0.63(0.44,0.91) | 1.05(0.86,1.28) | 0.93(0.78,1.1) | 0.38 |
| 8 | 8-71323529 | 71323529 | 0.63(0.44,0.91) | 1.05(0.86,1.28) | 0.93(0.78,1.1) | 0.38 |
| 8 | 8-71323530 | 71323530 | 0.63(0.44,0.91) | 1.05(0.86,1.28) | 0.93(0.78,1.1) | 0.38 |
| 8 | rs1460680 | 71326322 | **0.65(0.46,0.93)** | **1.06(0.87,1.28)** | 0.94(0.79,1.11) | 0.44 |
| 8 | 8-71326829 | 71326829 | 0.64(0.45,0.92) | 1.04(0.86,1.27) | 0.92(0.78,1.1) | 0.37 |
| 8 | 8-71327205 | 71327205 | 0.96(0.73,1.25) | 0.95(0.8,1.13) | 0.95(0.83,1.1) | 0.52 |
| 8 | rs2958367 | 71327441 | 1.07(0.88,1.3) | **0.99(0.87,1.11)** | 1.01(0.91,1.12) | 0.89 |
| 8 | rs2926706 | 71328483 | 1.03(0.88,1.22) | 0.98(0.88,1.09) | 0.99(0.91,1.09) | 0.87 |
| 8 | rs2926705 | 71328571 | 1.3(1.04,1.62) | 1(0.86,1.16) | 1.08(0.95,1.22) | 0.22 |
| 8 | 8-71329444 | 71329444 | 0.64(0.45,0.92) | 1.05(0.86,1.27) | 0.93(0.78,1.1) | 0.39 |
| 8 | rs2926703 | 71329548 | 1.07(0.88,1.3) | **0.99(0.87,1.11)** | 1.01(0.91,1.12) | 0.88 |
| 8 | rs2958368 | 71330166 | 1.3(1.04,1.62) | 1(0.86,1.16) | 1.08(0.95,1.22) | 0.22 |
| 8 | rs2926702 | 71330548 | **1.21(1,1.47)** | **0.88(0.77,1)** | 0.97(0.87,1.08) | 0.61 |
| 8 | 8-71332056 | 71332056 | 0.64(0.45,0.92) | 1.06(0.87,1.28) | 0.94(0.79,1.11) | 0.45 |
| 8 | rs2926701 | 71333158 | 1.05(0.87,1.28) | 1.01(0.89,1.14) | 1.02(0.92,1.13) | 0.67 |
| 8 | rs2977980 | 71334412 | 1.05(0.87,1.28) | 1.01(0.89,1.14) | 1.02(0.92,1.13) | 0.67 |
| 8 | rs4738083 | 71334618 | 0.64(0.45,0.92) | 1.04(0.86,1.27) | 0.93(0.78,1.1) | 0.38 |
| 8 | rs2977982 | 71334640 | 1.31(1.05,1.64) | 0.98(0.84,1.14) | 1.07(0.95,1.22) | 0.28 |
| 8 | rs2926700 | 71337782 | 1.3(1.04,1.62) | 0.99(0.86,1.15) | 1.08(0.95,1.22) | 0.23 |
| 8 | rs2926699 | 71338415 | 1.3(1.04,1.62) | 0.99(0.86,1.15) | 1.08(0.95,1.22) | 0.23 |
| 8 | rs2926698 | 71340385 | 1.3(1.04,1.63) | 1(0.86,1.16) | 1.08(0.96,1.22) | 0.21 |
| 8 | 8-71345356 | 71345356 | 0.96(0.74,1.25) | 0.97(0.82,1.15) | 0.97(0.84,1.12) | 0.67 |
| 8 | 8-71346440 | 71346440 | 0.64(0.45,0.92) | 1.07(0.88,1.29) | 0.94(0.79,1.12) | 0.48 |
| 8 | rs2290878 | 71348299 | 0.63(0.44,0.91) | 1.03(0.85,1.26) | 0.91(0.77,1.09) | 0.31 |
| 8 | rs1473409 | 71348381 | 1.05(0.86,1.28) | 1.01(0.9,1.15) | 1.02(0.92,1.14) | 0.65 |
| 8 | rs16936858 | 71348770 | 0.63(0.44,0.92) | 1.05(0.87,1.28) | 0.93(0.78,1.11) | 0.42 |
| 8 | 8-71349047 | 71349047 | 0.64(0.45,0.92) | 1.07(0.88,1.29) | 0.94(0.79,1.12) | 0.48 |
| 8 | rs16936861 | 71349197 | 1.3(1.04,1.62) | 1(0.86,1.16) | 1.08(0.95,1.22) | 0.22 |
| 8 | rs11993681 | 71351604 | 0.64(0.44,0.92) | 1.07(0.88,1.3) | 0.94(0.79,1.12) | 0.48 |
| 8 | rs10504471 | 71351682 | 0.96(0.74,1.25) | 0.98(0.82,1.16) | 0.97(0.84,1.13) | 0.72 |
| 8 | rs16936870 | 71351896 | 1.3(1.04,1.63) | 1(0.86,1.16) | 1.08(0.95,1.22) | 0.22 |
| 8 | rs12056770 | 71352704 | 0.64(0.44,0.92) | 1.07(0.88,1.3) | 0.94(0.79,1.12) | 0.48 |
| 8 | rs16936872 | 71354934 | 0.97(0.74,1.26) | 0.95(0.8,1.13) | 0.96(0.83,1.11) | 0.55 |
| 8 | rs4738085 | 71355729 | 0.64(0.44,0.92) | 1.07(0.88,1.3) | 0.94(0.79,1.12) | 0.48 |
| 8 | rs7003091 | 71356817 | 1.3(1.04,1.63) | 1(0.86,1.16) | 1.08(0.96,1.23) | 0.21 |
| 8 | rs16936875 | 71357568 | **0.65(0.46,0.93)** | 1.07(0.88,1.3) | 0.94(0.79,1.12) | 0.49 |
| 8 | rs7008415 | 71357745 | 1.05(0.87,1.28) | 1.02(0.9,1.15) | 1.03(0.92,1.14) | 0.63 |
| 8 | rs7825320 | 71358034 | 0.64(0.44,0.92) | 1.07(0.88,1.3) | 0.94(0.79,1.12) | 0.48 |
| 8 | rs35395175 | 71358612 | 1.36(1.05,1.77) | 0.95(0.82,1.1) | 1.03(0.91,1.17) | 0.64 |
| 8 | 8-71359134 | 71359134 | 0.64(0.44,0.92) | 1.07(0.88,1.3) | 0.94(0.79,1.12) | 0.48 |
| 8 | rs13277206 | 71359365 | 1.3(1.04,1.63) | 1(0.86,1.16) | 1.08(0.96,1.23) | 0.2 |
| 8 | 8-71359643 | 71359643 | 1.38(1.07,1.79) | 1.02(0.86,1.21) | 1.12(0.97,1.29) | 0.12 |
| 8 | rs17677348 | 71360101 | 1.05(0.89,1.24) | 0.99(0.89,1.1) | 1.01(0.92,1.1) | 0.9 |
| 8 | rs4738086 | 71360722 | 0.65(0.45,0.93) | 1.03(0.85,1.26) | 0.92(0.77,1.1) | 0.35 |
| 8 | rs56174599 | 71361023 | 0.97(0.75,1.26) | 0.95(0.8,1.13) | 0.96(0.83,1.11) | 0.55 |
| 8 | rs16936880 | 71362663 | 1.31(1.05,1.64) | **0.95(0.82,1.1)** | 1.04(0.92,1.18) | 0.48 |
| 8 | rs16936881 | 71362712 | 1.02(0.82,1.27) | 0.99(0.88,1.12) | 1(0.9,1.11) | 0.99 |
| 8 | rs16936884 | 71365569 | 0.97(0.75,1.26) | 0.95(0.8,1.13) | 0.96(0.83,1.11) | 0.55 |
| 8 | 8-71366610 | 71366610 | 0.64(0.44,0.92) | 1.07(0.88,1.3) | 0.94(0.79,1.12) | 0.48 |
| 8 | 8-71366973 | 71366973 | 1.02(0.82,1.27) | 1(0.88,1.13) | 1(0.9,1.12) | 0.94 |
| 8 | 8-71369750 | 71369750 | 0.64(0.44,0.92) | 1.07(0.88,1.3) | 0.94(0.8,1.12) | 0.51 |
| 8 | 8-71369952 | 71369952 | 0.64(0.44,0.92) | 1.07(0.88,1.3) | 0.94(0.79,1.12) | 0.51 |
| 8 | rs16936890 | 71369981 | 0.64(0.44,0.92) | 1.07(0.88,1.3) | 0.94(0.8,1.12) | 0.51 |
| 8 | rs16936891 | 71370334 | 0.64(0.44,0.92) | 1.07(0.88,1.3) | 0.94(0.79,1.12) | 0.51 |
| 8 | 8-71371363 | 71371363 | 0.64(0.44,0.92) | 1.07(0.88,1.3) | 0.94(0.79,1.12) | 0.51 |
| 8 | 8-71371774 | 71371774 | 0.64(0.44,0.92) | 1.07(0.88,1.3) | 0.94(0.79,1.12) | 0.51 |
| 8 | rs7824655 | 71372575 | 0.64(0.44,0.93) | 1.05(0.87,1.28) | 0.93(0.79,1.11) | 0.44 |
| 8 | rs6472517 | 71372885 | 1.02(0.82,1.27) | 1(0.88,1.13) | 1(0.9,1.12) | 0.94 |
| 8 | rs16936902 | 71373552 | 0.64(0.44,0.92) | 1.07(0.88,1.3) | 0.94(0.8,1.12) | 0.51 |
| 8 | 8-71376218 | 71376218 | 0.64(0.44,0.92) | 1.07(0.88,1.3) | 0.94(0.8,1.12) | 0.51 |
| 8 | rs12675772 | 71376259 | 1.36(1.04,1.76) | 0.97(0.84,1.13) | 1.05(0.92,1.19) | 0.46 |
| 8 | rs7015784 | 71376837 | 1.02(0.82,1.27) | 1(0.88,1.13) | 1(0.9,1.12) | 0.97 |
| 8 | rs13260060 | 71380914 | 1.32(1.05,1.64) | **0.95(0.82,1.1)** | 1.05(0.93,1.18) | 0.46 |
| 8 | 8-71384435 | 71384435 | 0.64(0.44,0.92) | 1.05(0.87,1.28) | 0.93(0.78,1.11) | 0.41 |
| 8 | rs16936907 | 71385319 | **1.12(0.8,1.56)** | 0.91(0.66,1.27) | 1.01(0.8,1.27) | 0.96 |
| 8 | rs6997367 | 71385775 | 1.04(0.84,1.29) | 1.01(0.88,1.15) | 1.02(0.91,1.14) | 0.79 |
| 8 | 8-71386985 | 71386985 | 0.64(0.44,0.92) | 1.07(0.88,1.3) | 0.94(0.79,1.12) | 0.5 |
| 8 | 8-71387746 | 71387746 | 0.64(0.44,0.92) | 1.07(0.88,1.3) | 0.94(0.79,1.12) | 0.5 |
| 8 | rs7837694 | 71387832 | 1.02(0.82,1.27) | 0.99(0.88,1.12) | 1(0.9,1.11) | 1 |
| 8 | rs17677919 | 71391161 | 0.98(0.8,1.19) | **0.95(0.85,1.07)** | 0.96(0.87,1.06) | 0.41 |
| 8 | 8-71394657 | 71394657 | 0.62(0.43,0.9) | 1(0.83,1.22) | 0.9(0.75,1.07) | 0.21 |
| 8 | rs7815765 | 71395285 | 1.05(0.88,1.24) | 0.98(0.88,1.09) | 1(0.91,1.09) | 0.94 |
| 8 | rs34017261 | 71395827 | 1.06(0.87,1.29) | 0.99(0.88,1.12) | 1.01(0.91,1.12) | 0.84 |
| 8 | rs16936913 | 71396438 | 0.64(0.44,0.92) | 1.07(0.88,1.3) | 0.94(0.79,1.12) | 0.5 |
| 8 | rs16936916 | 71397817 | 0.64(0.44,0.92) | 1.07(0.88,1.3) | 0.94(0.79,1.12) | 0.5 |
| 8 | rs16936918 | 71397874 | 0.64(0.44,0.92) | 1.07(0.88,1.3) | 0.94(0.79,1.12) | 0.5 |
| 8 | rs34957854 | 71399286 | 1.32(1.05,1.65) | 0.97(0.83,1.13) | 1.06(0.94,1.2) | 0.35 |
| 8 | rs16936921 | 71401846 | 0.64(0.44,0.93) | 1.05(0.87,1.28) | 0.93(0.79,1.11) | 0.44 |
| 8 | rs12675593 | 71402126 | 1.32(1.05,1.65) | 0.97(0.83,1.12) | 1.06(0.94,1.2) | 0.36 |
| 8 | rs10504472 | 71403032 | 0.96(0.74,1.26) | **0.98(0.82,1.16)** | 0.97(0.84,1.12) | 0.7 |
| 8 | rs35124548 | 71404064 | 1.36(1.05,1.77) | 0.95(0.82,1.11) | 1.04(0.91,1.18) | 0.56 |
| 8 | rs16936923 | 71405419 | 0.64(0.44,0.92) | 1.07(0.88,1.3) | 0.94(0.79,1.12) | 0.5 |
| 8 | rs16936924 | 71406139 | 0.64(0.44,0.92) | 1.07(0.88,1.3) | 0.94(0.79,1.12) | 0.5 |
| 8 | rs16936925 | 71406291 | 0.64(0.44,0.92) | 1.07(0.88,1.3) | 0.94(0.79,1.12) | 0.5 |
| 8 | rs11777095 | 71407373 | 1.36(1.05,1.77) | 0.96(0.83,1.12) | 1.04(0.92,1.19) | 0.51 |
| 8 | rs11780758 | 71407724 | 1.36(1.05,1.77) | 0.96(0.83,1.12) | 1.05(0.92,1.19) | 0.5 |
| 8 | rs11777228 | 71407844 | **1.36(0.97,1.89)** | 1.08(0.85,1.37) | 1.16(0.96,1.42) | 0.13 |
| 8 | rs6987924 | 71408463 | 1.29(1.03,1.62) | 1.01(0.87,1.18) | 1.09(0.97,1.24) | 0.16 |
| 8 | 8-71409519 | 71409519 | 0.64(0.44,0.92) | 1.07(0.88,1.3) | 0.94(0.79,1.12) | 0.49 |
| 8 | rs35006067 | 71411952 | 1.36(1.05,1.77) | 0.96(0.83,1.12) | 1.05(0.92,1.19) | 0.49 |
| 8 | rs4738088 | 71413558 | 0.64(0.44,0.93) | **1.05(0.87,1.28)** | 0.94(0.79,1.11) | 0.45 |
| 8 | rs4410938 | 71413638 | 1.36(1.05,1.77) | 0.96(0.83,1.12) | 1.05(0.92,1.19) | 0.5 |
| 8 | 8-71415883 | 71415883 | 1.4(1.08,1.81) | 0.98(0.82,1.17) | 1.09(0.94,1.26) | 0.23 |
| 8 | rs1014160 | 71416129 | 1.03(0.83,1.28) | 1(0.88,1.13) | 1.01(0.9,1.12) | 0.9 |
| 8 | rs16936926 | 71419312 | 1.03(0.83,1.28) | 1(0.89,1.14) | 1.01(0.91,1.13) | 0.85 |
| 8 | rs61606320 | 71422042 | 1.03(0.83,1.28) | 1(0.88,1.13) | 1.01(0.9,1.12) | 0.92 |
| 8 | rs12680132 | 71422481 | 1.05(0.88,1.24) | 0.97(0.88,1.08) | 0.99(0.91,1.09) | 0.88 |
| 8 | rs12676045 | 71422501 | 1.06(0.87,1.29) | 1(0.89,1.13) | 1.02(0.92,1.13) | 0.72 |
| 8 | rs10504473 | 71422886 | 1.06(0.87,1.29) | **0.98(0.87,1.11)** | 1(0.91,1.11) | 0.95 |
| 8 | rs10504474 | 71423014 | 1.31(1.05,1.63) | **0.95(0.82,1.1)** | 1.04(0.93,1.18) | 0.48 |
| 8 | rs7841047 | 71428532 | 1.03(0.83,1.28) | 0.99(0.87,1.12) | 1(0.9,1.11) | 0.99 |
| 8 | rs11986247 | 71428842 | 0.64(0.44,0.92) | 1.04(0.86,1.27) | 0.92(0.78,1.1) | 0.36 |
| 8 | rs16936937 | 71429208 | 0.64(0.44,0.92) | 1.04(0.86,1.27) | 0.92(0.78,1.1) | 0.36 |
| 8 | rs16936940 | 71429652 | 0.64(0.44,0.92) | 1.05(0.86,1.27) | 0.92(0.78,1.1) | 0.37 |
| 8 | rs35060705 | 71429966 | 1.36(1.05,1.77) | 0.98(0.84,1.14) | 1.06(0.93,1.21) | 0.39 |
| 8 | rs10504476 | 71430183 | 1.36(1.05,1.77) | 0.98(0.84,1.14) | 1.06(0.93,1.21) | 0.39 |
| 8 | rs7812666 | 71431011 | **1.05(0.87,1.26)** | 0.99(0.88,1.12) | 1.01(0.91,1.12) | 0.88 |
| 8 | rs12682284 | 71431433 | 1.36(1.05,1.77) | 0.98(0.84,1.14) | 1.06(0.93,1.21) | 0.38 |
| 8 | 8-71432485 | 71432485 | 1.36(1.05,1.77) | 0.98(0.84,1.14) | 1.06(0.93,1.21) | 0.37 |
| 8 | rs7823768 | 71433993 | 0.64(0.44,0.92) | 1.04(0.85,1.26) | 0.92(0.77,1.09) | 0.33 |
| 8 | rs16936942 | 71435922 | 0.97(0.74,1.26) | 0.95(0.8,1.13) | 0.96(0.83,1.1) | 0.54 |
| 8 | rs12056650 | 71439139 | 0.64(0.44,0.92) | 1.05(0.86,1.27) | 0.92(0.78,1.1) | 0.38 |
| 8 | 8-71439792 | 71439792 | 1.32(1.06,1.66) | 0.95(0.81,1.1) | 1.05(0.92,1.19) | 0.46 |
| 8 | 8-71439793 | 71439793 | 1.32(1.06,1.66) | 0.95(0.81,1.1) | 1.05(0.92,1.19) | 0.46 |
| 8 | rs35386521 | 71441088 | 1.37(1.05,1.78) | 0.96(0.83,1.12) | 1.05(0.92,1.2) | 0.47 |
| 8 | 8-71444992 | 71444992 | 0.64(0.44,0.92) | 1.05(0.86,1.27) | 0.92(0.78,1.1) | 0.38 |
| 8 | rs10435576 | 71447664 | 0.64(0.44,0.92) | 1.05(0.86,1.27) | 0.92(0.78,1.1) | 0.38 |
| 8 | rs10435577 | 71447665 | 0.64(0.44,0.92) | 1.05(0.86,1.27) | 0.92(0.78,1.1) | 0.38 |
| 8 | rs11777780 | 71448325 | 1.37(1.05,1.78) | 0.96(0.83,1.12) | 1.05(0.92,1.2) | 0.47 |
| 8 | rs13255359 | 71450398 | 1.32(1.06,1.66) | 0.95(0.81,1.1) | 1.05(0.92,1.19) | 0.48 |
| 8 | rs7012908 | 71451851 | 1.32(1.06,1.66) | 0.95(0.81,1.1) | 1.05(0.92,1.19) | 0.48 |
| 8 | rs12679364 | 71454590 | 1.06(0.87,1.29) | 0.98(0.87,1.11) | 1(0.9,1.11) | 0.96 |
| 8 | rs12544983 | 71455201 | 0.97(0.74,1.26) | 0.95(0.8,1.13) | 0.96(0.83,1.11) | 0.56 |
| 8 | 8-71455551 | 71455551 | 0.64(0.44,0.92) | 1.05(0.86,1.27) | 0.93(0.78,1.1) | 0.39 |
| 8 | rs12678300 | 71456291 | 1.32(1.06,1.66) | 0.95(0.81,1.1) | 1.05(0.92,1.19) | 0.48 |
| 8 | rs10957519 | 71456476 | 1.32(1.06,1.66) | 0.95(0.81,1.1) | 1.05(0.92,1.19) | 0.48 |
| 8 | 8-71456546 | 71456546 | 0.64(0.44,0.92) | 1.05(0.86,1.27) | 0.93(0.78,1.1) | 0.39 |
| 8 | rs28374075 | 71457835 | 1.32(1.06,1.66) | 0.95(0.81,1.1) | 1.05(0.92,1.19) | 0.48 |
| 8 | rs16936968 | 71458018 | 1.32(1.06,1.66) | 0.95(0.81,1.1) | 1.05(0.92,1.19) | 0.48 |
| 8 | rs16936971 | 71460066 | 0.64(0.44,0.92) | 1.05(0.86,1.27) | 0.93(0.78,1.1) | 0.39 |
| 8 | rs35050697 | 71460195 | 1.32(1.06,1.66) | 0.95(0.81,1.1) | 1.05(0.92,1.19) | 0.48 |
| 8 | rs60034031 | 71460503 | 1.32(1.06,1.66) | 0.95(0.81,1.1) | 1.05(0.92,1.19) | 0.48 |
| 8 | rs13276919 | 71461329 | 1.06(0.87,1.29) | 0.98(0.87,1.11) | 1(0.9,1.11) | 0.96 |
| 8 | rs11780251 | 71461909 | 1.32(1.06,1.66) | 0.95(0.81,1.1) | 1.05(0.92,1.19) | 0.48 |
| 8 | rs11780994 | 71462032 | 1.32(1.06,1.66) | 0.95(0.81,1.1) | 1.05(0.92,1.19) | 0.48 |
| 8 | 8-71462798 | 71462798 | 0.64(0.44,0.92) | 1.05(0.86,1.27) | 0.93(0.78,1.1) | 0.39 |
| 8 | rs12681491 | 71463008 | 1.31(1.05,1.63) | 0.96(0.82,1.12) | 1.06(0.93,1.2) | 0.39 |
| 8 | rs6472523 | 71465246 | 1.05(0.88,1.24) | 0.98(0.88,1.09) | 1(0.91,1.09) | 0.95 |
| 8 | 8-71469995 | 71469995 | 0.64(0.44,0.92) | 1.05(0.86,1.27) | 0.93(0.78,1.1) | 0.39 |
| 8 | rs10957520 | 71470666 | 1.32(1.06,1.66) | 0.95(0.81,1.1) | 1.05(0.92,1.19) | 0.48 |
| 8 | 8-71473658 | 71473658 | 0.97(0.74,1.26) | 0.96(0.81,1.13) | 0.96(0.83,1.11) | 0.57 |
| 8 | rs11989173 | 71475234 | 0.64(0.44,0.92) | 1.05(0.86,1.27) | 0.93(0.78,1.1) | 0.39 |
| 8 | 8-71476051 | 71476051 | 0.64(0.44,0.92) | 1.05(0.86,1.27) | 0.93(0.78,1.1) | 0.39 |
| 8 | rs6991199 | 71479151 | 1.06(0.87,1.29) | 0.98(0.86,1.11) | 1(0.9,1.11) | 0.98 |
| 8 | rs35229942 | 71480784 | 1.32(1.06,1.66) | 0.94(0.81,1.1) | 1.04(0.92,1.18) | 0.51 |
| 8 | 8-71481599 | 71481599 | 0.97(0.74,1.26) | 0.96(0.81,1.14) | 0.96(0.83,1.11) | 0.58 |
| 8 | 8-71482063 | 71482063 | 0.64(0.44,0.92) | 1.05(0.86,1.27) | 0.93(0.78,1.1) | 0.39 |
| 8 | rs11776606 | 71482851 | 1.32(1.06,1.66) | 0.94(0.81,1.1) | 1.04(0.92,1.18) | 0.51 |
| 8 | 8-71487669 | 71487669 | 0.64(0.44,0.92) | 1.05(0.86,1.27) | 0.93(0.78,1.1) | 0.39 |
| 8 | rs11776287 | 71489169 | 1.31(1.05,1.63) | 0.96(0.82,1.12) | 1.06(0.93,1.2) | 0.38 |
| 8 | 8-71490255 | 71490255 | 0.64(0.44,0.92) | 1.05(0.86,1.27) | 0.93(0.78,1.1) | 0.39 |
| 8 | rs11990849 | 71491009 | 0.64(0.44,0.92) | 1.05(0.86,1.27) | 0.93(0.78,1.1) | 0.39 |
| 16 | rs11076785 | 3730187 | 0.90(0.76,1.06) | **1.04(0.95,1.14)** | 1.01(0.93,1.09) | 0.89 |
| 16 | rs129968 | 3731262 | **0.97(0.84,1.12)** | **1.06(0.97,1.16)** | 1.03(0.96,1.12) | 0.4 |
| 16 | rs129963 | 3736148 | **1.15(1.0,1.32)** | **1.04(0.95,1.13)** | 1.07(0.99,1.15) | 0.085 |
| 16 | rs17136507 | 3740546 | **0.96(0.73,1.26)** | **1.09(0.93,1.28)** | 1.06(0.92,1.21) | 0.42 |
| 16 | rs886528 | 3751557 | **1.11(0.96,1.27)** | **0.94(0.86,1.02)** | 0.98(0.91,1.06) | 0.66 |
| 16 | rs11076786 | 3751597 | 1.41(1.14,1.73) | **0.97(0.84,1.12)** | 1.09(0.97,1.23) | 0.16 |
| 16 | rs130005 | 3768349 | 1.45(1.18,1.77) | 0.97(0.83,1.13) | 1.12(0.99,1.26) | 0.083 |
| 16 | rs130021 | 3772472 | **1.09(0.95,1.26)** | **0.97(0.89,1.06)** | 1.01(0.93,1.09) | 0.88 |
| 16 | rs130023 | 3773307 | **0.75(0.51,1.10)** | **1.13(0.90,1.42)** | 1.01(0.83,1.23) | 0.92 |
| 16 | rs12597046 | 3776167 | 1.21(0.89,1.66) | 0.97(0.83,1.13) | 1.01(0.88,1.16) | 0.89 |
| 16 | rs8046065 | 3778299 | **0.93(0.74,1.16)** | **1.02(0.89,1.16)** | 0.99(0.89,1.11) | 0.93 |
| 16 | rs62037859 | 3778901 | 0.89(0.76,1.05) | 0.97(0.88,1.08) | 0.95(0.87,1.04) | 0.26 |
| 16 | rs3789033 | 3779536 | 0.86(0.71,1.03) | **0.97(0.88,1.08)** | 0.94(0.86,1.03) | 0.21 |
| 16 | rs12599143 | 3790417 | 1.42(1.15,1.76) | 0.99(0.86,1.15) | 1.11(0.99,1.26) | 0.079 |
| 16 | rs11076787 | 3792777 | 1.02(0.84,1.23) | **1.07(0.96,1.19)** | 1.06(0.97,1.16) | 0.23 |
| 16 | rs11644593 | 3811287 | 1.40(1.13,1.75) | 0.97(0.83,1.13) | 1.09(0.96,1.24) | 0.18 |
| 16 | rs1296720 | 3813643 | **0.94(0.80,1.12)** | **1.08(0.97,1.20)** | 1.04(0.95,1.14) | 0.38 |
| 16 | rs2239317 | 3860741 | **1.37(1.12,1.68)** | 0.97(0.84,1.12) | 1.09(0.97,1.23) | 0.16 |
| 16 | rs2239318 | 3864938 | 1.64(1.24,2.16) | **0.97(0.84,1.12)** | 1.08(0.95,1.22) | 0.25 |

Bold font**, genotyped SNPs**
